# Effects of Particle Geometry for PLGA-Based Nanoparticles: Preparation and In Vitro/In Vivo Evaluation

**DOI:** 10.3390/pharmaceutics15010175

**Published:** 2023-01-03

**Authors:** Meryem Kaplan, Kıvılcım Öztürk, Süleyman Can Öztürk, Ece Tavukçuoğlu, Güneş Esendağlı, Sema Calis

**Affiliations:** 1 Department of Pharmaceutical Technology, Faculty of Pharmacy, Hacettepe University, Ankara 06100, Turkey; 2 Department of Pharmaceutical Technology, Faculty of Pharmacy, Süleyman Demirel University, Isparta 32260, Turkey; 3 Centre for Laboratory Animals Research and Application, Hacettepe University, Ankara 06100, Turkey; 4 Department of Basic Oncology, Hacettepe University Cancer Institute, Ankara 06100, Turkey

**Keywords:** nanoparticles, particle shape, anisotrop, human serum albumin, cellular uptake, biodistribution, drug delivery, PLGA

## Abstract

The physicochemical properties (size, shape, zeta potential, porosity, elasticity, etc.) of nanocarriers influence their biological behavior directly, which may result in alterations of the therapeutic outcome. Understanding the effect of shape on the cellular interaction and biodistribution of intravenously injected particles could have fundamental importance for the rational design of drug delivery systems. In the present study, spherical, rod and elliptical disk-shaped PLGA nanoparticles were developed for examining systematically their behavior in vitro and in vivo. An important finding is that the release of the encapsulated human serum albumin (HSA) was significantly higher in spherical particles compared to rod and elliptical disks, indicating that the shape can make a difference. Safety studies showed that the toxicity of PLGA nanoparticles is not shape dependent in the studied concentration range. This study has pioneering findings on comparing spherical, rod and elliptical disk-shaped PLGA nanoparticles in terms of particle size, particle size distribution, colloidal stability, morphology, drug encapsulation, drug release, safety of nanoparticles, cellular uptake and biodistribution. Nude mice bearing non-small cell lung cancer were treated with 3 differently shaped nanoparticles, and the accumulation of nanoparticles in tumor tissue and other organs was not statistically different (*p* > 0.05). It was found that PLGA nanoparticles with 1.00, 4.0 ± 0.5, 7.5 ± 0.5 aspect ratios did not differ on total tumor accumulation in non-small cell lung cancer.

## 1. Introduction

The National Nanotechnology Initiative (NNI, 2010) describes nanotechnology as “the understanding and control of matter at dimensions between approximately 1 and 100 nm, where unique phenomena enable novel applications” [1]. Over the past three decades, nanotechnology has emerged as a promising strategy to resolve the technological impasses incurred in various branches of science. Nanotechnology provides improvements of treatment efficiency by increasing the stability and solubility of encapsulated pharmaceuticals, contributing to transport across membranes, prolonging blood residence time, localizing drugs at the therapeutic site and exhibiting endosomal escape [2,3,4]. Moreover, targeting by affinity ligands facilitates the uptake of nanoparticles by the tumor cells themselves, allowing for prolonged tissue retention and increased cellular uptake [2]. It is also possible to design these systems so that controlled drug release can take place from the matrix, which may include both organic and inorganic materials [5]. It is important to note that nanoparticles clear rapidly from the systemic circulation via phagocytic uptake, mainly by the macrophages in the liver and spleen. A surfactant such as PEG or poloxamer can be used to modify the surface of nanoparticles to increase their stability in vivo [6,7,8]. Despite these potentials and the promising results obtained from cell culture and animal studies, approval rates of nanomedicines are relatively low. This comes from the differences between animal models and humans, complexity of diseases, and the heterogeneity of patients [3,4]. As it is well known, one of the most studied research areas of nanomedicines is cancer treatment. Solid tumors have enhanced permeability and retention (EPR) effects, which are defined by leaky vasculature and a lack of efficient drainage of the lymphatic systems in solid tumors, allowing nanomedicines to accumulate and be retained in the tumor. This phenomenon has been well accepted as one of the universal pathophysiological characteristics of solid tumors and considered during the development of nanocarriers for tumor targeting [9]. The particle size of the nanocarrier is accepted as the most important formulation parameter of nanomedicines to ensure EPR effects and is generally adjusted between 10 and 200 nm [10]. In addition to particle size, other designing parameters of nanomedicines such as shape, surface charge and composition should be considered specifically case by case and selected wisely to overcome the low clinical translation of nanomedicines as mentioned above. All these parameters are expected to have an impact on circulation the half-life, biodistribution, phagocytosis, cellular uptake, therapeutic effects and renal excretion of nanomedicine [11,12,13]. The shape of the nanoparticle (NP) seem to have a direct impact on cellular uptake. Although there are some studies that suggest the opposite, nanoparticles with high aspect ratio such as rods and tubes show lower cellular uptake than sphericals [14]. Gratton et al. [15] synthesized nanoparticles larger than 100 nm and showed that rods have the highest uptake, followed by spheres, cylinders, and cubes. On the other hand, they found that spheres have an appreciable advantage over rods under 100 nm [16]. The shape of the nanocarrier also has an effect on blood circulation time. Obtaining longer blood circulation time with nanocarriers is always one of their main purposes for successful drug delivery. While spherical nanoparticles showed higher accumulation in organs responsible for clearance, other shapes such as rods, elliptical carriers and worms were able to escape phagocytosis at a high rate [17].

In the concept of this study, the in vitro and in vivo effects of particle shape as a crucial formulation parameter for passive tumor targeting were investigated on the A549 cell line and non-small cell lung cancer animal model. Firstly, the idea of developing nonspherical particles was formed by taking inspiration from pathogens (virus, bacteria, etc.), which mostly have a nonspherical shape and have features including an escape from the phagocytic cells, a long circulation time and high cellular uptake [18,19]. Nonspherical nanocarrier preparation methods can be classified briefly as non-wetting templates, microfluidic systems, template assemblies and film stretching. The film stretching method is a simple, versatile, inexpensive and high-throughput method. In the film stretching method, different nonspherical shapes can be fabricated by the immobilization of polymeric particles in a polyvinyl alcohol (PVA) film, and heating the film above the glass transition temperature of polymeric particles, followed by stretching to deform the particles [12,20,21,22,23]. In this study, rod and elliptical disk polymeric nanoparticles were prepared starting from spherical ones using the film stretching method adapted from the literature [22,24,25].

Poly (lactic-co-glycolic acid, 50:50) (PLGA) was selected as the polymer for formulations due to its advantages such as FDA approval for biomedical applications, biodegradability, biocompatibility, providing sustained and controlled release, and easy functionalization for stealth and targeting purposes [26,27]. Endogenous protein human serum albumin (HSA) was used as the model payload for comparing encapsulation efficiency and drug release profiles. It’s aim was to evaluate the effects of the geometry of PLGA nanoparticles on in vitro characteristics and accumulation at tumor sites.

## 2. Materials and Methods

### 2.1. Materials

PLGA Resomer^®^ RG 503 (50:50 ratio of PLA:PGA, Mw:35.000 Da) was purchased from Boehringer Ingelheim (Ingelheim am Rhein, Germany). Human serum albumin, polyvinyl alcohol (PVA) (average Mw 30,000–70,000 Da), and methylthiazolyltetrazolium (MTT) were obtained from Sigma-Aldrich Co. (St. Louis, MO, USA). Methoxy-poly(ethylene glycol)-b-poly(lactide-co-glycolide)-FKR560 copolymer (mPEG-PLGA-FKR560, Mw~5–10 kDa) was purchased from Akina, Inc. (West Lafayette, IN, USA). Dulbecco’s Modified Eagles (DMEM), fetal bovine serum (FBS), and penicillin/streptomycin solutions were supplied from Biochrom AG (Berlin, Germany). Sodium dodecyl sulfate (SDS) and dimethylformamide (DMF) were purchased from J.T. Baker (Deventer, The Netherlands). L929 and A549 cells were purchased from the American Type Culture Collection (ATCC, Rockville, MD, USA). All Type I water was of analytical grade (Milli-Q Plus System–Millipore/Millipore Corporation, Burlington, MA, USA).

### 2.2. Preparation of Spherical PLGA Nanoparticles

PLGA NPs were prepared by W/O/W double emulsion solvent evaporation method by two-step homogenization procedure [28]. Briefly, 750 µL of the inner aqueous phase (2% PVA solution) was added to the organic phase (PLGA in dichloromethane, 2.5 mL) on the magnetic stirrer, it was then emulsified in the ice bath through the ultrasonic probe (Sonopuls HD 4200, Bandelin, Berlin, Germany) at 40% amplitude and pulse at 0.5 s. The first emulsion was added on to 0.5% PVA aqueous solution (10 mL) under continuous stirring using magnetic stirrer and emulsified by sonication. Afterwards, dichloromethane was evaporated under the fume hood and finally, collection of the NPs was carried out by centrifugation (Universal 320, Hettich, Tuttlingen, Germany) at 13,000 rpm for 45 min. Nanoparticles obtained were washed thrice using type I water for removing PVA residue. HSA loaded nanoparticles were prepared using the same method described above in which 100 µg HSA was dissolved in aqueous inner phase.

### 2.3. Preparation of Differently Shaped Nanoparticles

**Figure 1:**
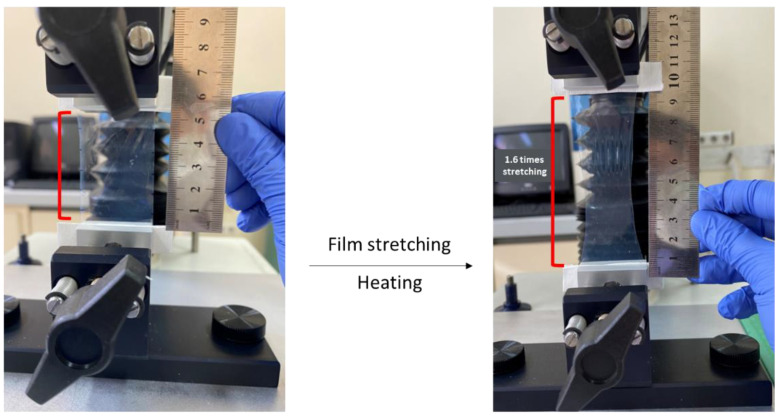
Production of differently shaped nanoparticles by film stretching method using texture analyzer.

Rod and elliptical disk-shaped nanoparticles were prepared starting from spherical nanoparticles using the modified film stretching method adapted from literature [22,25]. Firstly, 5% PVA solution containing glycerol at 2% concentration was prepared for film production, glycerol was added to ensure the elasticity and homogeneous mixture of the film. Then, the solution was filtered through a 0.45 µm cellulose acetate membrane filter. Afterwards, spherical nanoparticles were added into the filtered solution at a concentration of 0.05–0.1%, this mixture was poured into the 15 × 8 × 1.5 cm template (film solution thicknesses approx. 3–3.5 mm) and dried for 16 h in drying-oven (Memmert GmbH, Schwabach, Germany) at 45 °C. Spherical nanoparticles containing dried films were cut into 3.7 cm × 7.5 cm sections. Obtained film was inserted to the tensile grip apparatus of the analyzer and stretching was performed at a rate of 0.3–0.5 mm/s (Figure 1).

After applying a heater (Philips, HP4668/22) set at 100 °C for 3 min to the film, the stretching process (while heat application continued) was performed. The aspect ratio (AR), defined as ratio of the nanoparticles’ major axis length to the minor axis length, was controlled by limiting the extent of stretching [29]. The process was terminated at the point where the film was stretched 1.6 (for rod shaped) and 2.24-fold (for elliptical disk shaped). The Texture Analyzer XT-Plus (Stable Micro Systems, Surrey, UK) was used for generating of nonspherical nanoparticles first time in literature [22]. Finally, stretched films were dispersed in 5 mL of ultra-pure water on the magnetic stirrer. Nanoparticles were collected by centrifugation at 6000 rpm for 20 min at 18 °C and washed 4 times with type I water to remove PVA residue from the surface of the particles.

### 2.4. In Vitro Characterization of Blank and HSA-Loaded PLGA Nanoparticles

#### 2.4.1. Determination of Particle Size, Polydispersity Index, and Zeta Potential

Mean particle size and polydispersity index (PDI) of nanoparticles were determined at 25 °C by dynamic light scattering, using Malvern Instruments Zetasizer Nano-ZS (Malvern Instruments, Malvern, UK). Each sample was dispersed in deionized water and placed in disposable measurement cells and measured in triplicates. The analysis was performed at a scattering angle of 90° with a material refraction index of 1.33 [30]. Nanoparticles were also characterized in terms of zeta potential using the same instrument (n = 3).

#### 2.4.2. Morphology of Differently Shaped PLGA Nanoparticles

Transmission electron microscopy (TEM; Tecnai G2 Spirit BioTwin CTEM, FEI, Hillsboro, OR, USA) was used to investigate the surface morphology and to confirm the particle size of the prepared PLGA NPs. Sufficient volume of nanoparticle dispersion was placed on carbon-coated copper grid and allowed to air dry for about 2 min. The samples were visualized at accelerating voltage of about 120 kV [31].

#### 2.4.3. Encapsulation Efficiency (EE)

The encapsulation efficiencies were determined indirectly (detection of unloaded HSA in the supernatants). Firstly, HSA-loaded PLGA NPs were centrifuged at 13,500 rpm for 45 min at 20 °C, then the supernatant was collected. Afterwards, samples were analyzed by a micro Bicinchoninic Acid (BCA) protein assay kit (Takara Bio, Shiga, Japan) and procedures were carried out following manufacturer’s instructions. This is a very sensitive colorimetric method to quantify proteins at very low concentrations (0–200 µg/mL) [32]. Protein concentrations were determined by generating a standard curve (range by 5–200 µg/mL) based upon using the BSA standard solutions. In the first step, 100 μL of a working solution was added to 100 μL of the sample, then the mixture was incubated in drying-oven at 37 °C for 60 min, and absorbances of the solutions were evaluated at 562 nm at final step.

EE was determined by calculating the percentage of drug encapsulated into the nanoparticles using the following standard formula. All measurements were conducted in triplicate and the results were reported as the mean ± SD.

(1) $$\%\;\mathrm{EE}=\frac{\left(\mathrm{the}\;\mathrm{total}\;\mathrm{amount}\;\mathrm{of}\;\mathrm{drug}\;\mathrm{added}\;\mathrm{for}\;\mathrm{the}\;\mathrm{preparation}\right)-\left(\mathrm{the}\;\mathrm{amount}\;\mathrm{of}\;\mathrm{drug}\;\mathrm{in}\;\mathrm{supernatant}\right)}{\left(\mathrm{the}\;\mathrm{total}\;\mathrm{amount}\;\mathrm{of}\;\mathrm{drug}\;\mathrm{added}\;\mathrm{for}\;\mathrm{the}\;\mathrm{preparation}\right)}\times 100$$

#### 2.4.4. Drug Release Study

The release studies of the NPs were conducted in triplicates using sample and separate (SS) method under sink conditions [33]. Eppendorf tubes (NPs containing 10 µg HSA approx.) dispersed in a total volume of 1 mL phosphate buffer (PBS, pH 7.4) were placed in a horizontal shaker water bath (Memmert, Schwabach, Germany) at 37 °C with a constant agitation of 80 strokes/min. At predetermined time intervals, the samples were centrifuged (13500 rpm for 30 min) and the amount of HSA in the supernatant determined by micro-BCA assay, using the procedure described Section 2.4.3.

### 2.5. Cell Culture Studies

#### 2.5.1. Effect of Nanoparticle Shape on Safety

Cytotoxicity of spherical, rod, and elliptical disk-shaped PLGA nanoparticles were evaluated on mouse fibroblast cell line (L929) as recommended by U.S. Pharmacopeial Convention (USP 26) and ISO 10993-5 guidelines for safe study [34,35,36]. Cell viability was evaluated using MTT assay. L929 cells were cultured in DMEM medium containing 10% FBS, 1% penicillin, and streptomycin solution and incubated in a 5% CO_2_ humidified atmosphere at 37 °C. The cells were seeded in 96-well plate at 2 × 10^4^ cells/well and incubated for 24 h (n = 4). Then, nanoparticles between 10 and 500 µM concentration range were applied to the cells and incubated for 24 and 48 h. At the end of the incubation periods, 5 mg/mL MTT solution (25 μL) was added to the wells to obtain formazan crystals and incubated for 4 h. Afterwards, 80 μL DMF:water (45:55) mixture containing SDS at a concentration of 0.23 g/mL was added to each well to dissolve the formazan crystals. After overnight incubation, the optical density (OD) was measured by a microplate reader (SpectraMax Plus, Molecular Devices) at 570 nm. The cell viability was calculated by the given formula below:

(2) $$\mathrm{Viability}\;\%=\frac{\mathrm{Mean}\;\mathrm{OD}\;\mathrm{of}\;\mathrm{samples}}{\mathrm{Mean}\;\mathrm{OD}\;\mathrm{of}\;\mathrm{control}\;\mathrm{group}}\times 100$$

#### 2.5.2. Cellular Uptake Study

A human non-small cell lung cancer (NSCLC) cell line (A549) was used for cellular uptake studies. Fluorescent labelled and PEGylated copolymer (mPEG-PLGA-FKR560, max. absorbance 562 nm) was used to prepare differently shaped nanoparticles to track and evaluate formulations easily in terms of cellular uptake. Cells were grown in high-glucose DMEM (w/o L-Glutamine) medium supplemented with 10% fetal bovine serum and 1% penicillin–streptomycin maintained in a humidified atmosphere with 5% CO_2_ at 37 °C. A549 cells were seeded a 24-well plate at 10 × 10^4^ cells/well and incubated for 24 h. Nanoparticle formulations (spherical, rod, elliptical disk) were resuspended in cell culture medium and applied to each well at a concentration of 200 µg/mL. After 8 h of incubation, the cells were collected and the median fluorescence intensity (MFI) of these cells was measured by flow cytometry. For fluorescence microscopy analysis, round glass coverslips were placed in the wells. Then, similarly to flow cytometry analysis, cells were seeded, formulations were applied and incubated for 8 h. At the end of incubation, cells were stained with DAPI (300 nM; Sigma, St. Louis, MO, USA) and examined under fluorescence microscopy (Olympus America Inc, Center Valley, PA). Finally, images were processed using the ImageJ software (NIH Image, Bethesda, MD, USA) [37].

### 2.6. In Vivo Biodistribution of Nanoparticles

All the experiments and handling of animals were performed following approval by Kobay A.S. Animal Research and Ethics Committee (Approval number:2019/354). Male CD-1 nude mice (23–24 g, 6–8 weeks old) were maintained in IVCs at 22 ± 3 °C, 55% relative humidity and under a 12 h dark/light cycle. Mice were allowed free access to food and water. Xenograft tumor model was developed by the subcutaneous injection of 1 × 10^7^ A549 cells in 100µL PBS coated with matrigel (1 mg/mL) to the dorsal flank of mice. When the tumor diameter reached about 0.5 cm, mice were separated into 4 groups to apply spherical-shaped nanoparticles (SN) (n = 3), rod-shaped nanoparticles (RN) (n = 3), elliptical disk-shaped nanoparticles (EDN) (n = 3), and polymer solution (n = 3) as control group. Nanoparticles (mPEG-PLGA-FKR560; 18 mg/mL) were injected intravenously through lateral tail vein of mice. Mice were sacrificed and dissected at 16 h post-injection. Fluorescent intensity of tissues was recorded using Newton 7.0 (Vilber, Collégien, France), under 580 nm filter. Mean fluorescence intensity of nanoparticles was calculated using the ImageJ Fiji program (Madison, WI, USA) [38].

### 2.7. Statistical Analysis

All experiments were performed in triplicate. Results were presented as mean ± SD (standard deviation). One-way analysis of variance (ANOVA) with Tukey test, Mann–Whitney U test, Kruskal–Wallis test, and Student’s *t* test were used for multiple comparisons in cell culture assays and in vivo studies via GraphPad Prism 9 (GraphPad Software Inc., San Diego, CA, USA).

## 3. Results and Discussion

### 3.1. In Vitro Characterization of Blank and HSA-Loaded PLGA Nanoparticles

#### 3.1.1. Determination of Particle Size, Polydispersity Index, and Zeta Potential

**Figure 2:**
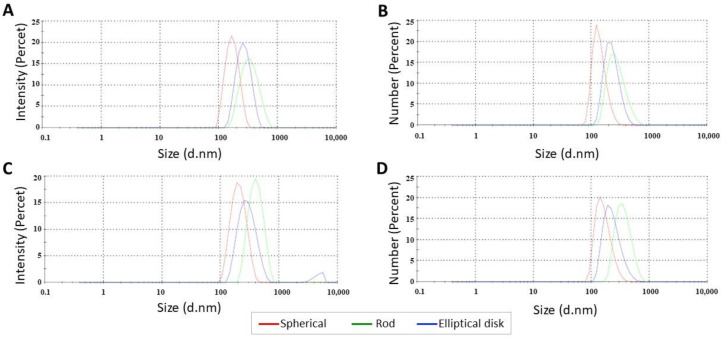
Particle size distribution of (**A**,**B**) blank and (**C**,**D**) HSA loaded nanoparticles.

SN were first formulated successfully by the double emulsion method and differently shaped PLGA NPs were prepared by the film stretching method (n = 3) as described previously in Section 2.3. The results of this study showed that the change in nanoparticle shape can be controlled by the film stretching ratio. Although the developed stretching process allows for us to obtain both a wide range of sizes and aspect ratios of nanoparticles [39], there are still various challenges. Blank SNs, RNs, and EDNs exhibited sizes of 163.2 ± 0.702, 312.9 ± 5.401 (AR: 4.0 ± 0.5), and 251.4 ± 4.737 nm (AR: 7.5 ± 0.5), respectively (Table 1, Figure 2). The change in aspect ratio may have caused an increase in the average particle size of RNs and EDNs, and similar studies are available in the literature [25,40,41].

Since the measurement principle of the nanosizer device is optimized for spherical particles, the results obtained for nonspherical NPs were considered as an indicator of the shape alteration. Small particle size (<400 nm), which is the most important requirement for EPR-based passive tumor targeting, was provided for all generated nanoparticles [42]. The mean particle size increase due to HSA loading on the nanoparticles was not statistically significant (*p* > 0.05). For all NP formulations, the PDI values were below 0.2 (range from 0.06 to 0.22), which revealed the monodispersity of formulations. A PDI value of less than 0.2 is generally considered suitable for drug delivery systems with a narrow particle size distribution and evaluated as highly monodisperse [43,44,45]. It is thought that the increase in the PDI value of nonspherical nanoparticles is due to the production method and multiple centrifugation processes to remove the film. All formulations had zeta potential values between −0.6 mV and −17.2 mV. As known, a zeta potential absolute value higher than 25 mV is required for high colloidal stability in drug delivery systems, which prevents aggregation by repelling the same charged particles in the dispersion medium [46]. While the zeta potential of SNs and EDNs formulations was anionic (absolute value >10 mV), RNs were approximately neutral (absolute value < 10 mV) [47]. It was observed that the zeta potential of nanoparticles with different geometries prepared with the same polymer decreased compared to spherical nanoparticles (*p* < 0.05) [48]. Although the spherical shape is advantageous in terms of physicochemical stability, the zeta potential value of nonspherical nanoparticles can also be improved using optimized production methods or polymer modifications. The surface charge of nanoparticles was not an extensively studied parameter in studies based on shape, so the available data were limited in terms of comparison [13]. In addition, unlike sphericals, it is not possible to mention a homogeneous charge distribution in nonspherical NPs. Therefore, more sensitive methods may be required for zeta potential measurement.

#### 3.1.2. Morphology of Differently Shaped PLGA Nanoparticles

**Figure 3:**
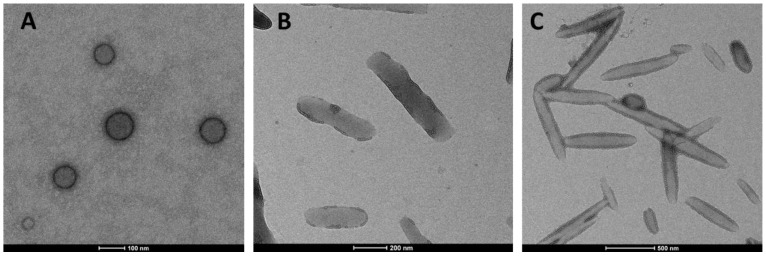
TEM images of differently shaped blank PLGA nanoparticles ((**A**) Spherical; (**B**) Rod; (**C**) Elliptical disk).

Confirmation of the rod and elliptical disk shape formations was performed by TEM analysis. As shown in Figure 3, the ultrastructural morphology of the nanoparticles was spherical, rod and elliptical disk, respectively, and they appeared to have a smooth surface with a size range compatible with nanosizer measurements.

#### 3.1.3. Encapsulation Efficiency

The encapsulation efficiencies of HSA in NPs were calculated by an indirect method and were found to be between 86.2% and 91% (*p* > 0.05). From an economic point of view, the efficiency of encapsulation is extremely important, especially when the active substance is very expensive, as is typical for macromolecular drugs [49]. Due to the addition of the water-soluble drug molecule (HSA) to the inner aqueous phase, the encapsulation efficiency was high for all three shapes, as expected. The amount of PLGA in the intermediate organic phase has a crucial influence on the efficiency of encapsulation. A high EE% value in drug delivery systems is desirable in terms of keeping the polymer load given to the body at a low level [49].

#### 3.1.4. Drug Release Study

**Figure 4:**
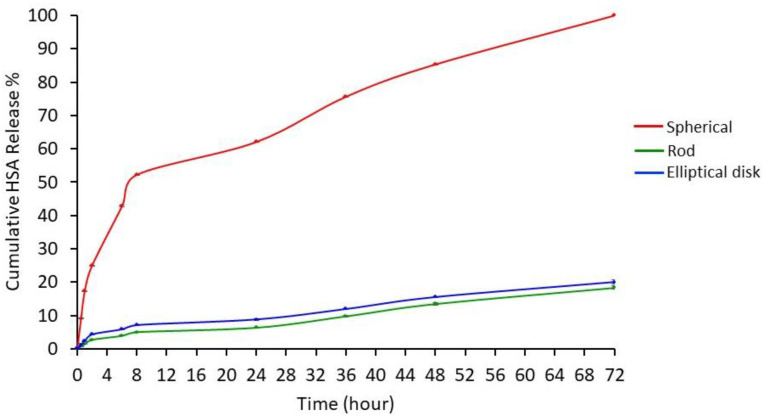
HSA release profiles from differently shaped nanoparticles at pH 7.4 (n = 3). Data were expressed as the mean ± SD, error bars were not visible due to SD values being too small.

According to the release study results, 100% of the HSA was released in 72 h from SNs. The cumulative release results of rod and elliptical disk-shaped nanoparticles at the end of 72 h were found to be 18.4% and 20.1%, respectively (Figure 4). Biodegradable polymer-based NPs such as PLGA enable drug release at a constant rate through diffusion. However, in the same conditions, nonspherical particles may indicate a different drug release profile than spherical ones due to having a different surface area and porosity [19,25,50].

Release test results were analyzed using the KINETDS 3.0 program and it was determined that the release profiles of the nanoparticles for all three geometries were in accordance with the Weibull release kinetics. The Weibull model is suitable for examining the release kinetics of both immediate- and prolonged-release drug delivery systems [51]. When the profiles were evaluated, it was observed that HSA is released from spherical nanoparticles higher than rod and elliptical disk-shaped nanoparticles (*p* < 0.05). As described in the nanoparticle preparation section, rod and elliptical disk-shaped nanoparticles were prepared from spherical nanoparticles. So, drug-loaded spherical nanoparticles might have been bearing HSA on the surface. The rod and elliptical disk-shaped nanoparticle preparation methods had additional steps including adding nanoparticles into the PVA (5%) solution, stretching in the PVA film, and several washing steps to remove the PVA residue. There might be HSA loss during washing steps which causes a decrease in HSA release [52].

### 3.2. Cell Culture Studies

#### 3.2.1. Effect of Nanoparticle Shape on Safety

**Figure 5:**
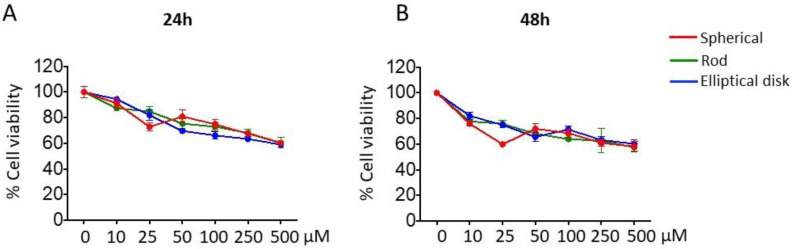
Cell viability profiles of blank SNs, RNs, and EDNs for (**A**) 24 h and (**B**) 48 h.

Time- and dose-dependent-based MTT studies were performed to investigate whether the change in shape could affect the safety of PLGA nanoparticles or not. When the 24 h results for nanoparticles were examined (Figure 5A), cell viability was between approximately 87% and 60% depending on the concentration, and for 48 h results (Figure 5B), cell viability was approximately 79% and 59% in nanoparticles. There was no significant difference dependent on shape in viability values for both time points that evaluated concentration range (*p* > 0.05).

#### 3.2.2. Based on Different Shapes, Uptake of the Nanoparticles may Change

**Figure 6:**
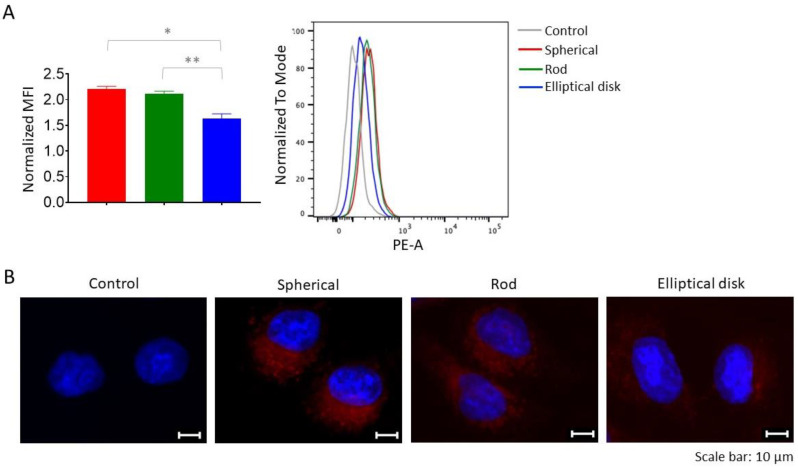
(**A**) Mean fluorescent intensities (MFI) of nanoparticles were measured by flow cytometry and the representative flow cytometry histograms were given (* *p* < 0.05 spherical compared with elliptical disk, ** *p* < 0.05 rod compared with elliptical disk). (**B**) Representative fluorescent microscopy images of A549 cells treated with SNs, RNs, and EDNs, cells were stained using DAPI (blue) for nucleus. Red fluorescence shows PLGA (FKR560).

One of the critical parameters affecting cellular uptake and the intracellular traffic of nanoparticles is geometry [53]. The uptake of nanoparticles by A549 cells was evaluated by the flow cytometry and fluorescent microscopy. The uptake of spherical and rod-shaped nanoparticles was comparable with EDNs (2.206 ± 0.043, 2.121 ± 0.037, 1.632 ± 0.076 normalized MFI for SNs, RNs, and EDNs, respectively (*p* < 0.05) Figure 6).

According to the results of flow cytometry analysis (Figure 6A), the highest uptake was observed in SNs, followed by RNs and EDNs. When Figure 6B is examined, it can be concluded that the ranking of differently shaped nanoparticles (SN > RN > EDN) was the same for the qualitative analysis. It has been known that rod or tubular-like nanoparticles are taken by cells more slowly than SN, and this pattern reveals an advantage when it comes to macrophages, which provide longer blood circulation time for nonspherical particles [54]. Similarly to the literature, the higher uptake rate of SNs by cells compared to RNs and EDNs can be explained by the contact angle. The internalization of RN and EDN nanoparticles by cells depends on contact angle, whereas it is independent in SNs [24,54,55]. In addition, the contact of nonspherical nanoparticles with cells with its short or long sides has an effect on the cellular uptake rate. Particularly, particles with a high AR value (~20) are aligned with their long axis parallel to the cell membrane, and internalization would be slower. Mathematical models have been developed to explain this condition [54].

### 3.3. Biodistribution and Targeting Efficiency of Nanoparticles

**Figure 7:**
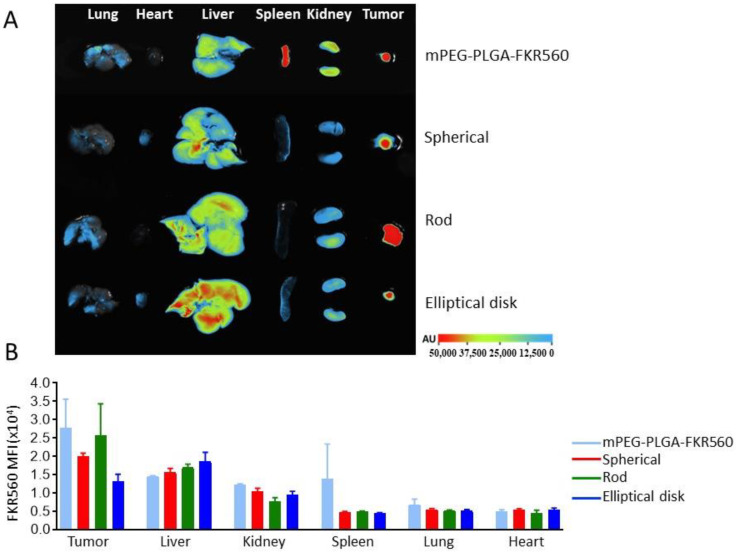
Biodistribution of differently shaped nanoparticles in NSCLC tumor-bearing CD-1 nude mice. (**A**) Fluorescence images of tumor and major organs (**B**) MFI values were obtained from tumor and organs after FKR560-labelled PLGA solutions and FKR560-labelled PLGA-based NP applications.

Longer blood circulation time is necessary for drug targeting. It has been known that nanoparticle geometry has an important effect on the biodistribution of nanoparticles [56,57]. Since nonspherical nanoparticles have an ability to escape from the reticuloendothelial system, their blood residence time is longer compared to spherical ones [54]. Within the scope of this experiment, the effect of nanoparticle shape on the biodistribution in tumor-developed mice was investigated (Figure 7).

Although it was assumed that the accumulation of RNs in tumor tissues was higher than SNs and EDNs from fluorescence images and calculated MFI values, differences could not be found that were significant statistically (*p* > 0.05) (Figure 7). For the same as tumor tissues, after a 16 h application the difference of accumulation for all 3 shaped nanoparticles in the liver, kidneys, spleen, lungs, and heart were found to be statistically insignificant (*p* > 0.05). In the concept of this experiment animals were sacrificed and dissected at 16 h post-injection. The experiment was performed at one time point to analyze the total accumulation. It would be beneficial to analyze at different time points to clarify the accumulation rate.

## 4. Conclusions

Differently shaped NPs with unique geometrical, physical and chemical properties have been designed in recent years using advanced nanofabrication techniques and a large variety of biocompatible materials. Despite the various methods used to fabricate nonspherical nanoparticles, it has been reported that reliable methods for the characterization of these particles are limited. Hence, comparing the experimental results with other related studies and predictions about the effect of shapes might be critical. Shape can be considered a tool to modify the nanocarrier for different purposes including the efficient accumulation of nanoparticles at the tumor microenvironment. There are nonspherical nanoparticle fabrication methods which have different approaches; the method should be selected according to material, active substance, and preferred shape. In the concept of this work, the fabrication and optimization of PLGA nanoparticles in rod and elliptical disk forms was carried out successfully using a modified film stretching method. As experienced, there are a few drawbacks for this method including that the film could not be stretched homogenously, which causes yield loss. As a critical stability parameter of colloidal systems, zeta potential might decrease depending on aspect ratio increases, which may be considered a weakness of nonspherical nanoparticles. The release profile could also be influenced by particle shape independently of polymer, active substance, and fabrication method. Detailed analyses are required to clarify geometry-dependent change in the release profiles of nanoparticles. Although the most appreciated feature of nanoparticles with a high aspect ratio is known to be their long blood circulation time resulting in higher tumor accumulation, this hypothesis could alter depending on tumor model, nanoparticle type, and the aspect ratio range. So, in this study we compared spherical, rod (AR:4.0 ± 0.5), and elliptical disk (AR:7.5 ± 0.5)-shaped nanoparticles in terms of tumor accumulation in an NSCLC animal model. Differences in tumor accumulation ratios are found to be insignificant statistically. Experimental evidence showed that a comparative study on specific disease models is required before designing nanocarriers. In summary, particle shape/geometry plays an important role in the rational design of particulate systems for drug delivery and targeting.

## Figures and Tables

**Table 1:** Particle size, polydispersity index, zeta potential and encapsulation efficiency of nanoparticles (n = 3).

Formulation	Particle Size(nm)	Aspect Ratio	Polydispersity Index (PDI)	Zeta Potential (mV)	EE (%)
**SN**	163.2 ± 0.7	1.0	0.032 ± 0.70	−17.20 ± 0.15	-
**RN**	312.9 ± 5.4	4.0 ± 0.5	0.079 ± 0.03	−4.08 ± 0.16	-
**EDN**	256.8 ± 4.7	7.5 ± 0.5	0.067 ± 0.01	−9.52 ± 0.34	-
**SN-HSA**	193.3 ± 0.9	-	0.055 ± 0.03	−14.50 ± 0.51	91
**RN-HSA**	375.3 ± 3.5	-	0.112 ± 0.03	−0.62 ± 0.03	86.3
**EDN-HSA**	294.3 ± 1.6	-	0.215 ± 0.01	−12.90 ± 0.70	86.2

(SN: Spherical nanoparticle, RN: Rod nanoparticle, EDN: Elliptical disk nanoparticle, HSA: Human serum albumin).

## Data Availability

Not applicable.
